# A multichannel color filter with the functions of optical sensor and switch

**DOI:** 10.1038/s41598-021-02453-2

**Published:** 2021-11-25

**Authors:** Yuan-Fong Chou Chau, Chung-Ting Chou Chao, Hung Ji Huang, Sy-Hann Chen, Tsung Sheng Kao, Hai-Pang Chiang

**Affiliations:** 1 Centre for Advanced Material and Energy Sciences, Universiti Brunei Darussalam, Tungku Link, Gadong, BE1410 Brunei Darussalam; 2 Department of Optoelectronics and Materials Technology, National Taiwan Ocean University, Keelung, 20224 Taiwan, ROC; 3 Taiwan Instrument Research Institute, National Applied Research Laboratories, Hsinchu, 300 Taiwan, ROC; 4 Department of Electrophysics, National Chiayi University, 600, Chiayi, Taiwan, ROC; 5 Department of Photonics & Institute of Electro-Optical Engineering, College of Electrical and Computer Engineering, National Yang Ming Chiao Tung University, Hsinchu, 300 Taiwan, ROC

**Keywords:** Nanoscience and technology, Optics and photonics

## Abstract

This paper reports a multichannel color filter with the functions of optical sensor and switch. The proposed structure comprises a metal–insulator–metal (MIM) bus waveguide side-couples to six circular cavities with different sizes for filtering ultra-violet and visible lights into individual colors in the wavelength range of 350–700 nm. We used the finite element method to analyze the electromagnetic field distributions and transmittance properties by varying the structural parameters in detail. The designed plasmonic filter takes advantage of filtering out different colors since the light-matter resonance and interference between the surface plasmon polaritons (SPPs) modes within the six cavities. Results show that the designed structure can preferentially select the desired colors and confine the SPPS modes in one of the cavities. This designed structure can filter eleven color channels with a small full width at half maximum (FWHM) ~ 2 nm. Furthermore, the maximum values of sensitivity, figure of merit, quality factor, dipping strength, and extinction ratio can achieve of 700 nm/RIU, 350 1/RIU, 349.0, 65.04%, and 174.50 dB, respectively, revealing the excellent functions of sensor performance and optical switch, and offering a chance for designing a beneficial nanophotonic device.

## Introduction

Surface plasmon polaritons (SPPs) are the amplification of electromagnetic (EM) waves since the interaction of incident photons with conduction electrons of metal nanoparticles (MNPs) on the surface of the metal-dielectric boundary^1–7^. The light-matter interaction depends on the dimension, volume, aspect of the MNPs, and the origin and constitution of the dispersion material^8–10^. SPPs waves solve the problem of the light diffraction limit and could confine the light within nanometer scale; consequently, they have broad-ranging applications of SPPs wave in integrated optical circuits (IOCs) and optical devices^11–15^, such as absorbers^16,17^, modulators^18,19^, splitters^20^, amplifiers^21,22^, switches^23,24^, filters^25,26^, sensors^27–31^ and so forth. Among them are metal–insulator–metal (MIM) waveguide-cavity-based devices with long propagation distances (1–40 μm), strong EM wave confinement, inexpensive production, low loss, and ease of fabrication and integration have attracted considerable interest and consideration^32–35^.

By comprehending the nature outlines of the light-matter coupling of changed MNP geometries and the corresponding material effect, new approaches have been exploited for cautious spectroscopic inspecting. Each variation can be manipulated by various spectroscopic techniques, which result in imaging events and sensing applications. From successful SPP mode monitoring through spectroscopy, new nanophotonic devices, including color sensors and devise sensor elements, have been investigated and developed^36^. Color filters can cope with precise wavelengths of concern or filter ultra-violet and visible light into separate colors or wavelengths. Nowadays, color filters are essential in many imaging devices, such as display units of computerized systems, digital projectors, digital photography, organic light-emitting diodes, etc.^37,38^. Researchers are consecutively looking for a scheme to create low-price, compact, and transmission effectual color filters to improve the traditional pigment-based printing. Recent advancements in manufacturing MNP-based structures have exhibited a method to surmount the diffraction and reach nano-size resolution^39^. One such technique can utilize plasmonic cavities merging SPPs, which can couple incident light into EM modes transferring onto the MIM surface^40,41^.

Recently, several research groups exploited the plasmonic color filters based on SPPs through different MIM-cavity-based configurations. In Ref.^37^, Zhang and coauthors proposed a multiband four-channel color filter employing an elliptical-shaped cavity and obtained the maximum sensitivity of 608 nm/RIU and figure of merit (FOM) of 105.02. Diest et al.^38^ experimentally and numerically demonstrated that arranged two slits into the waveguide and distinguished three primary colors, i.e., red, green, and blue. Combining six μ-ring cavities, Butt et al.^42^ partitioned the visible light into six colors and claimed that the highest sensitivity and figure of merit in mode 1 (i.e., at the largest resonance wavelength) could reach 700 nm/RIU and 191.6 1/RIU, respectively. However, the transmittance spectral of these articles show a higher full width at half maximum (FWHM) and a smaller dipping strength, which influence the resolution in color filtering and the performance of refractive index (RI) detecting.

This work proposed a novel and simple frame of the plasmonic color filter based on MIM-cavity configuration employing one bus waveguide side-coupled to six circular cavities. We used the two-dimensional (2-D) finite element method to analyze the EM field distributions and transmittance resonance modes by varying the structural parameters in detail. The introduction of six circular cavities near the MIM bus waveguide acting as resonators can remarkably improve the sensing performance because of their excellent optical features in the EM field confinement and low Ohmic loss. In addition, the resonance modes or channels found in the cavities have a significant impact on the coupling efficiency between the bus waveguide and resonance cavities, which is less discussed before and is needed to investigate further. The designed plasmonic filter can separate eleven color channels with a small full width at half maximum (FWHM) ~ 2 nm in the ultraviolet and visible ranges, which cannot attain in other reported articles.

Furthermore, six air cavities can preferentially select the desired colors (or wavelengths) and confine the SPPS modes in one of the cavities. In addition, the designed optical filter can function as an optical sensor and an all-optical switch, which possess the merit of excellent sensing performance and a high extinction ratio. Compared to the reported color filters, the proposed structure is straightforward and is rarely investigated before. The achieved plasmonic sensor is ideal for color detection design and can apply in nanophotonic devices, such as RI sensors and optical switches, and the range of the detected RI is available for testing RI materials.

## Structure model, simulation method, and fundamental

**Figure 1:**
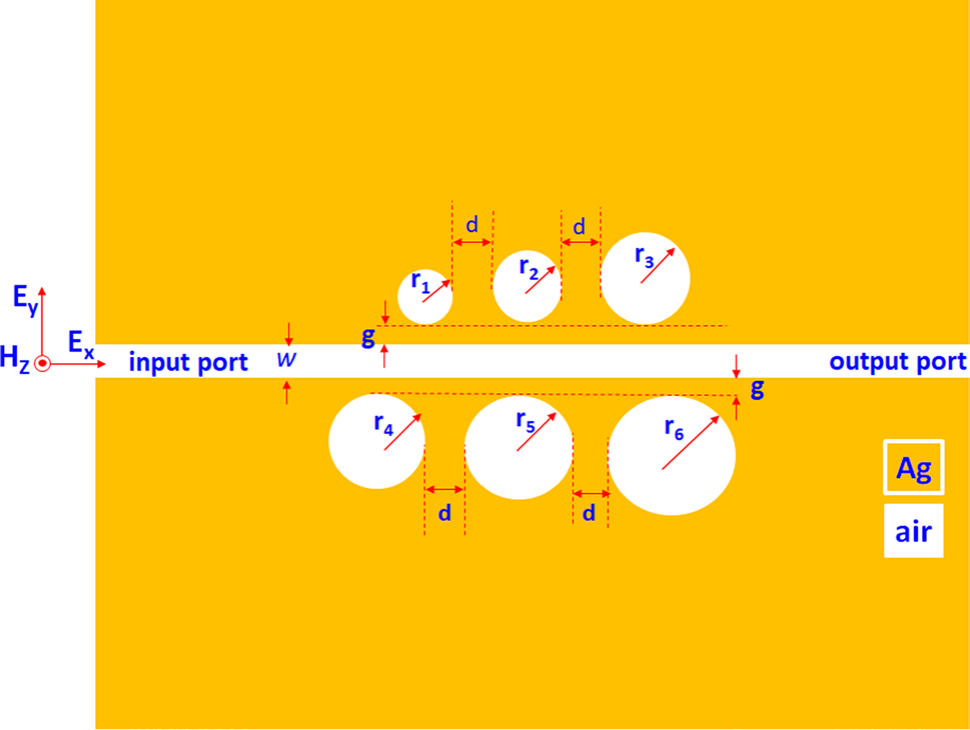
Top view of the proposed color filter, consisting of a MIM bus waveguide side-coupled with six circular cavities.

Figure 1 shows the top view of the investigated color filter, consisting of a MIM bus waveguide side-coupled with six circular cavities (with the radius of *r*_*1*_*, r*_*2*_*, r*_*3*_*, r*_*4*_*, r*_*5*_*,* and *r*_*6*_) at both sides of the bus waveguide. We indicated the structural parameters in Fig. 1, i.e., the width of the bus waveguide is w, the gap between the bus waveguide and the six circular ring-shaped cavities is *g*, the space between each circular cavity is *d*, respectively. In Fig. 1, the golden- and white-colored regions signify the silver and insulator medium (air with the RI of *n* = 1.00), respectively. We used a FEM-based commercial software, COMSOL Multiphysics, to calculate the transmittance spectrum and EM field distribution. The perfect matched layer boundary condition to form a border to avoid EM wave’s reflections and extra-fine triangular meshing for the discretization of the simulation domains. The proposed color filter in the *z*-direction is infinite compared to the x- and y-axes. Thus, the simulation model is a 2-D one since a three-dimensional (3-D) model can obtain similar results in simulations^43^ and experiments^44^. Besides, the 2-D simulations can save the computer resources^45^ without sacrificing the calculation precision. A TM-polarized EM wave with incident wavelength ranging in 350–700 nm coupled with the fundamental SPP mode^46–48^ into the bus waveguide's input port^49,50^.

Among all metals, silver (Ag) and gold (Au) have been the top choices for researchers due to numerous advantages^51^, providing satisfactory plasmonic response at optical frequencies. We choose silver as the plasmonic material because it can generate an EM wave response within the ultra-violet and visible range and exhibits the most negligible Drude damping, the small imaginary part of the relative permittivity, lower cost-consuming, and less power consumption. In addition, the other stable metals (e.g., Au, copper, or Pt) can also use in simulations and fabrication. The relative permittivity (ε_m_) of Ag can characterize by the Drude model^52^

1 $${\upvarepsilon }_{\mathrm{m}}\left(\upomega \right)={\upvarepsilon }_{\infty }-\frac{{\upomega }_{\mathrm{p}}^{2}}{{\omega }^{2}+i\omega \gamma }$$

where ω is the frequency, ω_p_ = 9.10 eV, ε_∞_ = 3.7 and γ = 18 meV are bulk plasma frequency, infinite dielectric constant, and electron collision frequency, respectively. The transmittance (T) can calculate as T = P_out_ (output power)/P_in_ (input power). The resonance modes in the individual cavity will occur when the SPPs from the bus waveguide coupled to the circular pits match the resonance condition. If Δφ = 2π*j* (*j* is an integer), the resonance wavelength (λ_res_) can be expressed by^53,54^

2 $${{\uplambda }_{\mathrm{res}}=\frac{2{L}_{eff}{Re(n}_{\mathrm{eff}})}{j-\frac{\varphi }{2\pi }} \left(j=\mathrm{1,2},3\dots \right)}$$

here, *j* denotes the order of the standing wave resonance, *L*_eff_ represents the cavity’s effective length, φ stands for the phase shift, and Re(*n*_eff_) is the effective RI’s real part. *n*_eff_ can describe as:

3 $${Re(n}_{\mathrm{eff}})={\left({\varepsilon }_{metal}+{\left(\frac{k}{{k}_{0}}\right)}^{2}\right)}^\frac{1}{2}$$

k = 2π/λ and *k*_0_ are the wave vector in material and free space, respectively.

Sensitivity (S) and figure of merit (FOM) are two essential factors for sensing design. We use *S* = Δλ/Δ*n* (nm/RIU, RIU is a RI unit) and FOM = S/FWHM to calculate the S and FOM, where Δλ is the λ_res_ shift of transmittance, and Δ*n* is the difference in the RI corresponding to λ_res_. Full width at half-maximum (FWHM) can define as the bandwidth value connected to the left and right of the half-high position of the transmittance spectrum. Besides, we can obtain quality (Q) factor using λ_res_/FWHM.

The proposed color filter can manufacture using stripping and electric beam lithography (EBL) processes^55^, which can imprint custom pictures with sub-10 nm resolution^51^. The fabrication of the proposed color filter is a straightforward process. First, a thin layer of Ag can deposit on a silica substrate followed by EBL patterning of the design. Then, one can use wet etching (e.g., diluted nitric acid) to eliminate the undesired Ag through lift-off. However, the purpose of this article does not to concentrate on the fabricating process. As a substitute, some potential papers that focus on this theme are advised^56–58^.

## Design of a multichannel plasmonic color filter

**Figure 2:**
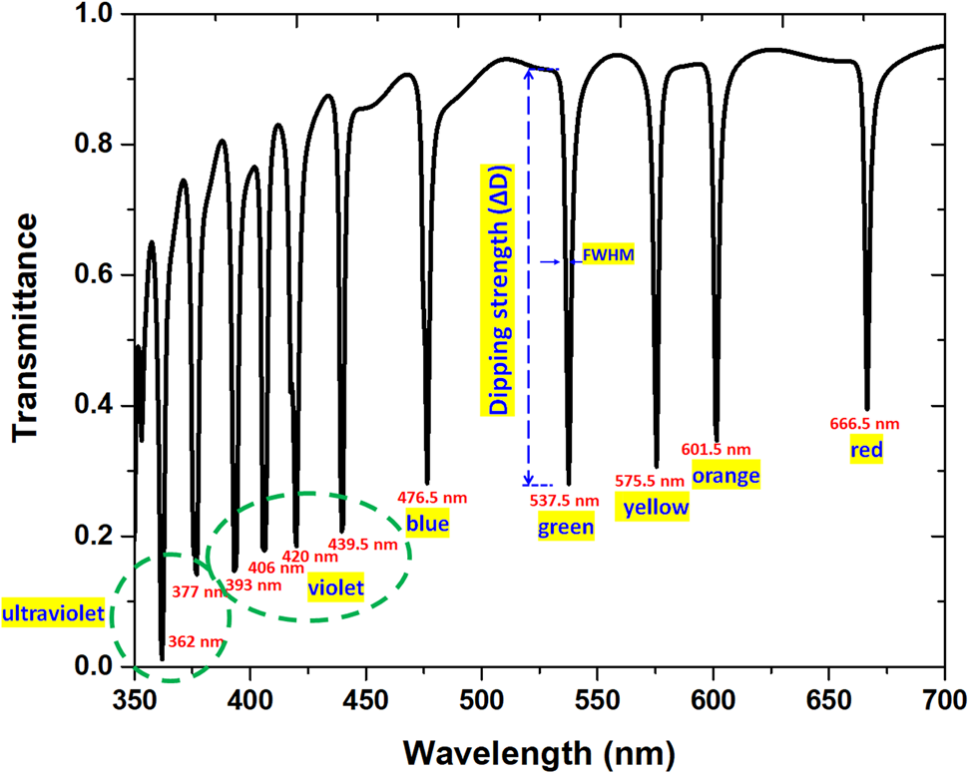
Transmittance spectrum of the proposed color filter structure ranging in 350–700 nm.

For simplicity, we do not discuss optimizing geometrical parameters on the proposed color filter but directly provide the default values, as shown in Table 1. Figure 2 depicts the transmittance spectrum of the SPPs mode for the proposed structure. Here, we define the dipping strength (ΔD) as the difference between the maximum and minimum transmittance (i.e., ΔD = (T_max_ − T_min_) × 100%)^59^.

**Table 1:** Default geometrical parameters of the proposed color filter.

*r*_*1*_ (nm)	*r*_*2*_ (nm)	*r*_*3*_ (nm)	*r*_*4*_ (nm)	*r*_*5*_ (nm)	*r*_*6*_ (nm)	*w* (nm)	*g* (nm)	*d* (nm)
80	100	120	132	140	160	100	40	100

In Eq. (2), λ_res_ is proportional to *L*_eff_, while the effective RI (*n*_eff_) and mode integer *j* are the same. One can easily detect color if the λ_res_ is recognized. The ultraviolet and visible light impinges into the input end of the bus waveguide side-coupled to the six circular cavities when the resonance condition in the circular cavities is satisfied. As shown in Fig. 2, eleven sharp transmittance dips corresponding to the different resonance modes (i.e., mode 1 to mode 11 or channel 1 to channel 11) distribute in the range of visible and ultraviolet, which accords with red (λ_res_ = 666.5 nm), orange (λ_res_ = 601.5 nm), yellow (λ_res_ = 575 nm), green (λ_res_ = 537.5 nm), blue (λ_res_ = 476.5 nm), violet (λ_res_ = 439, 420, 406, 393 nm), and ultraviolet (377 and 363 nm) colors, respectively. Therefore, one can establish the detection of those specific colors ranging in ultraviolet and visible. These SPP modes are due to the cavity plasmon resonance (CPR) and surface plasmon resonance (SPR) arising from the coupling effect between the six circular cavities and the bus waveguide^60^. The interference of SPR and CPR cause the multichannel SPPs modes between bus waveguides and circular cavities^8,61,62^, leading to eleven available SPPs modes in the wavelength of 350–700 nm. We found that CPR plays a pivotal role in offering more plasmon resonance in the proposed color filter system. The obtained transmittance dips possess a more profound dipping strength (∆D) ranging in 53.28–66.71% and a narrower FWHM of ~ 2 nm compared to the reported articles (e.g.,^37,42^), both beneficial to the resolution of the color filter and the performance of RI sensor. It is worth noting that four and two channels ranging in violet and ultraviolet lights appeared in the proposed color filter, which the previous articles cannot attain (e.g.,^37,38,42^). Violet and ultraviolet rays are prioritized in this scenario as recent works studied the application of the violet light in various cases such as surgical management^63^, dental bleaching^64^, fungal study^65^, and forensic science^66^. This remarkable feature of the designed filter has led to potential applications in the medical field.

**Figure 3:**
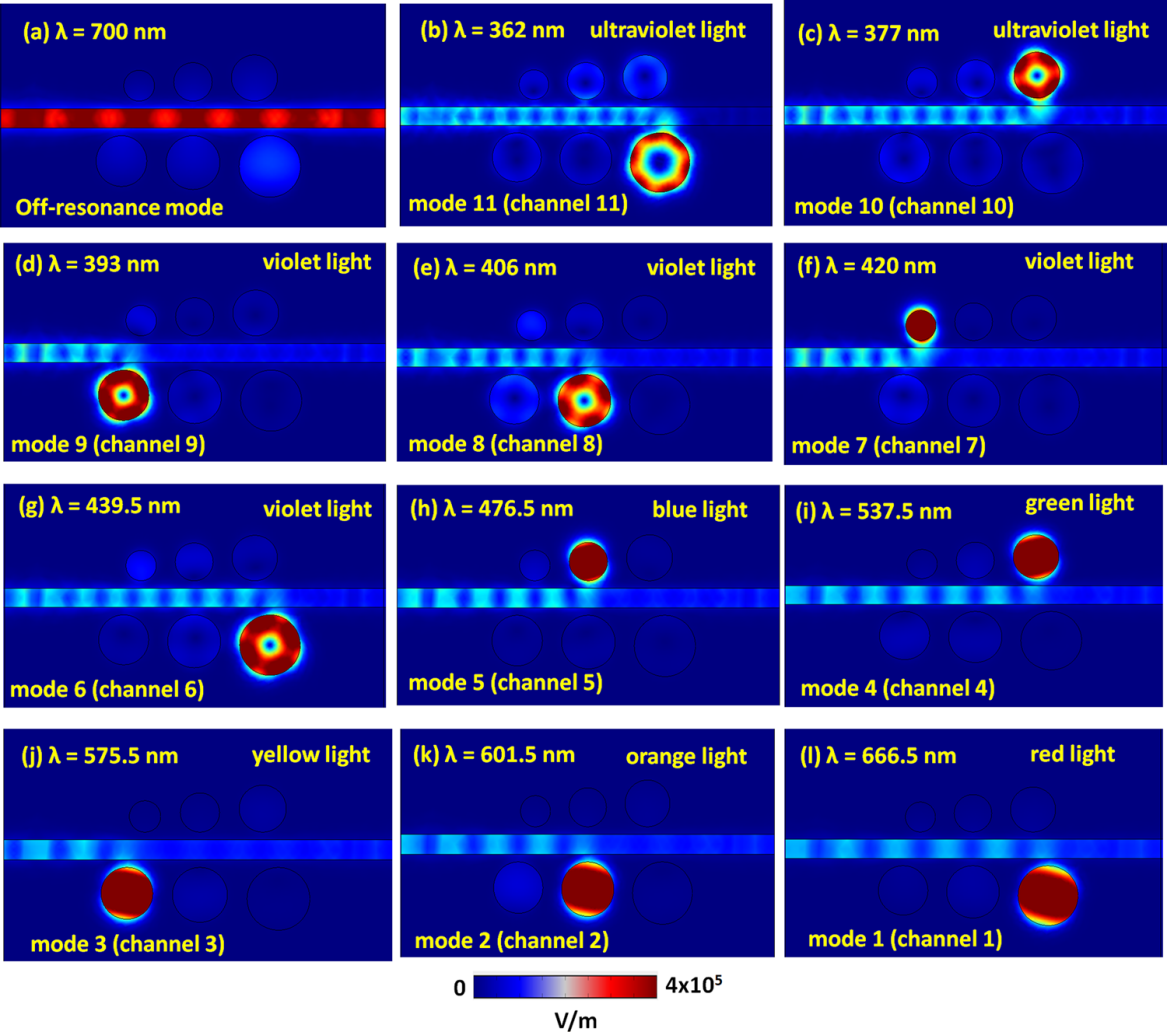
Truncate views of electric field intensity (|E|) distributions at one of the off-resonance wavelengths (λ_res_ = 700 nm) and eleven resonance wavelengths (λ_res_ = 362, 377, 393, 406, 420, 439.5, 476.5, 537.5, 575.5, 601.5 and 666.5 nm) from channel 1 to channel 11 (i.e., mode 1–mode 11), respectively.

When the incident EM wave satisfies the resonance condition in the optical filter system, the SPPs’ energy delivered from the bus waveguide to the six cavities through near field coupling. It allows constructing a stable standing wave mode in the six cavities, which can productively engineer the profile of the transmittance spectrum. To comprehend the physical mechanism of resonance modes that occurred in the investigated filter system, Fig. 3a–l illustrate the electric field intensity (|E|) distributions at one of off-resonance wavelengths (λ_res_ = 700 nm) and eleven resonance wavelengths (λ_res_ = 362, 377, 393, 406, 420, 439.5, 476.5, 537.5, 575.5, 601.5 and 666.5 nm) from channel 1 to channel 11 (i.e., mode 1 to mode 11), respectively. The wavelengths of EM waves with different phases associate with the energy and establish the apparent color. When λ_res_ at off-resonance mode (Fig. 3a), the SPPs wave hardly stays in the six cavities, showing that the SPPs wave in the bus waveguide has a constructive interference, while in the cavity has a destructive interference.

On the contrary, plasmon modes are restricted predominantly to the left side of the bus waveguide and the cavities at λ_res_. As a result, SPPs cannot propagate on the right side of the bus waveguide, which accords with the observation of low transmittance (i.e., long ΔD) at λ_res_. Based on the simulations (not shown here for simplicity), the proposed structure cannot form multiple channels and filter functions if the proposed system only exists in one cavity_._ As observed in Fig. 3b–l, different resonance modes confine in the specific cavity, which offers eleven channels of λ_res_, respectively. Thus, the circular cavities can behave as the Fabry-Pérot cavities in the optical filter system, and one of six cavities can effectively trap the incident light at λ_res_. According to the |E| field patterns, the SPPs wave can couple well because of the constructive interference between the bus waveguide and one of the six circular cavity resonators, exhibiting a significant CPR effect. The |E| field enhancement of the SPPs modes in the six circular cavities reveals an excellent light-matter interaction. Besides, the dipole effect, i.e., positive–negative charge pairs, may induce along the periphery of the six circular cavities, forming the vigorous confinement of SPPs and providing destructive interference at the right end of the bus waveguide. Thus, the evident transmittance dips could attain in Fig. 2.

## Optimization of the parameters of *g* and *d*

The SPPs modes originating from the proposed color filter are due to the coupling effect between the bus waveguide and the six circular cavities, significantly influenced by the structural size. For ease of fabrication, the structural parameters are less as possible. In the proposed color filter, w is fixed at 100 nm to promise that the TM mode can propagate in the bus waveguide. According to the FEM simulations (not shown here for simplicity), we can get the different colors of λ_res_ by modifying suitable radii of circular cavities (i.e., r_1_–r_6_) based on the structural parameters listed in Table 1. In other words, we can attain the desired color in terms of wavelength ranging in ultraviolet and visible by choosing an appropriate size of circular cavities. Therefore, we further examine the other two parameters in our simulations, i.e., *g* and *d*.

**Figure 4:**
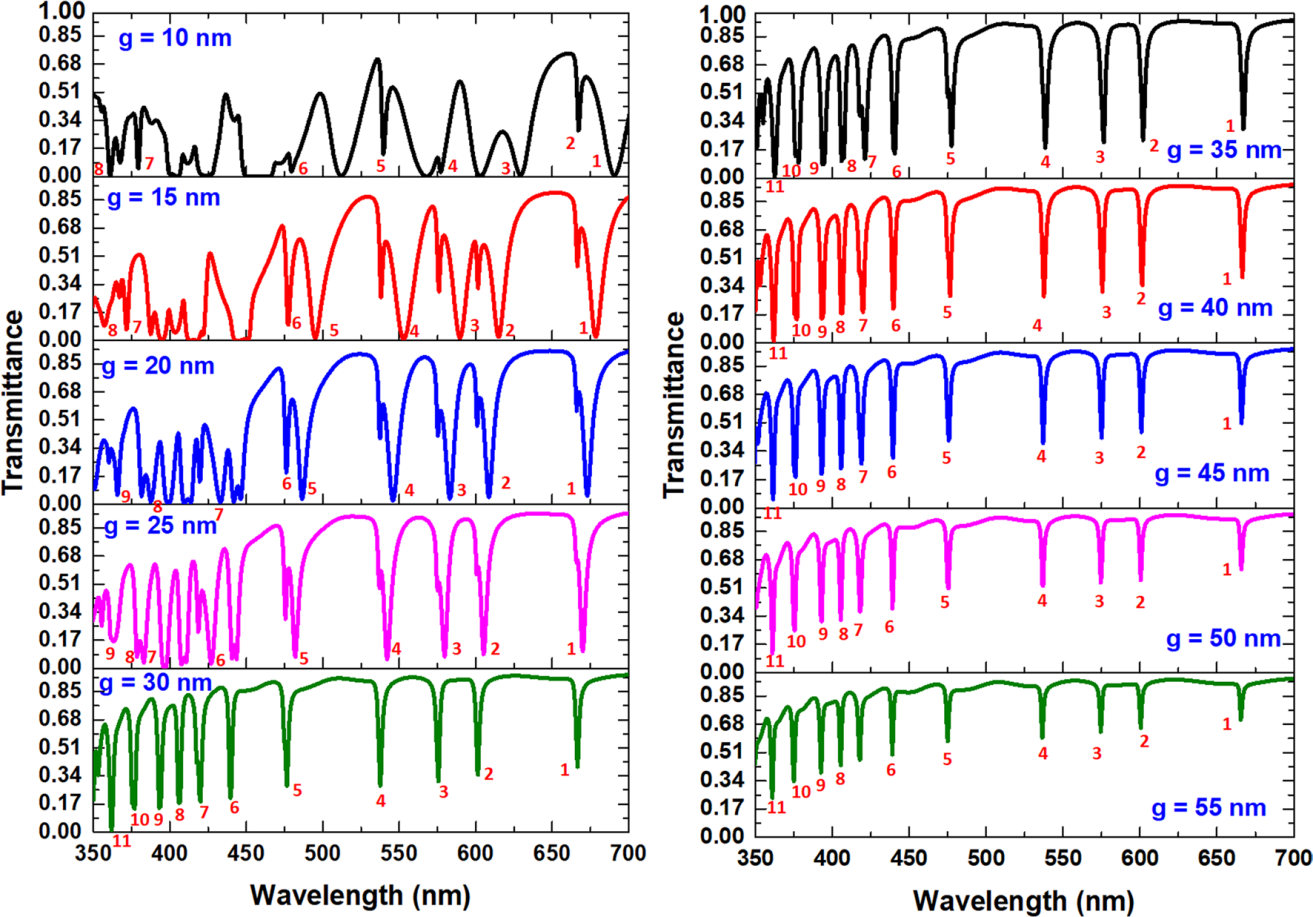
Transmittance spectrum of the proposed color filter for different values of *g* from 10 to 55 nm, with an interval of 5 nm.

First, Fig. 4 depicts the transmittance spectrum of the proposed color filter for different values of *g* from 10 to 55 nm, with an interval of 5 nm. The other structural parameters are the same as used in Table 1. We numbered the available operation channels depending on the sharper resonance dip, smaller FWHM, and more extended ∆D in the inset of this figure. As seen, the transmittance dips decrease with the increasing *g*. The transmittance has a different line shape to the variation of *g* because of their different coupling effect between bus waveguide and side-coupled cavities. Each channel has a slight resonance dip at the left side of the saddle point when *g* ≤ 25 nm and vanishes when *g* ≥ 30 nm. Furthermore, when g ≤ 25 nm, the resonance dips are not sharp enough in the short wavelength range (i.e., ranging in 350–450 nm). It is worth noting that the transmittance curves show a strong oscillation since the more substantial coupling effect is when *g* ≤ 25 nm. Besides, the ∆D and FWHM will significantly reduce when *g* ≥ 50 nm due to the more negligible coupling effect of a more significant *g*. Based on Fig. 4, the acceptable values of *g* are in the range of 25–45 nm based on the viewpoint of the workable channel numbers, transmittance line-shape, ∆D, and FWHM.

**Figure 5:**
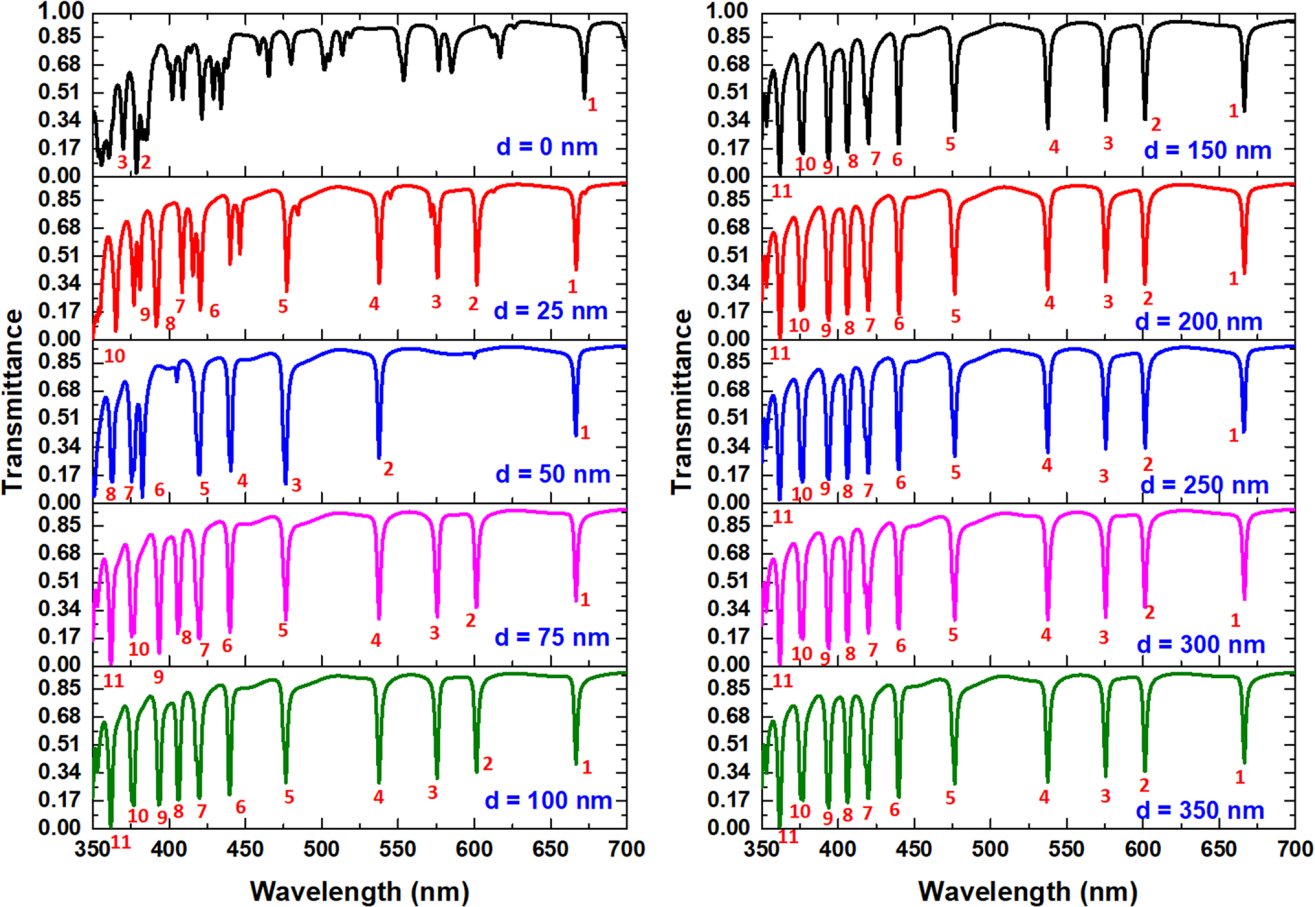
Transmission spectra as a function of *d* variation of the proposed color filter.

Successively, we show the variation influence of *d* on the transmittance spectrum in Fig. 5. As shown, a short ∆D and a fierce oscillation of transmittance line-shape arising from the violent coupling effect between the connected cavities when *d* = 0 nm. Nevertheless, the λ_res_ remains the same values with increasing *d* since the less interaction between adjacent holes when *d* ≥ 25 nm. In general, a mistake during fabrication has a severe influence on the efficacy of plasmonic devices. A slight error in different structural sizes can significantly alter the sensitivity performance. Fortunately, the shift of λ_res_ by varying *d* is less sensitive to the change of *d*, implying that the robust fabrication of the proposed color filter. Based on Fig. 5, the workable values of *d* can choose in the range of 25–350 nm.

## Application as a refractive index sensor

**Figure 6:**
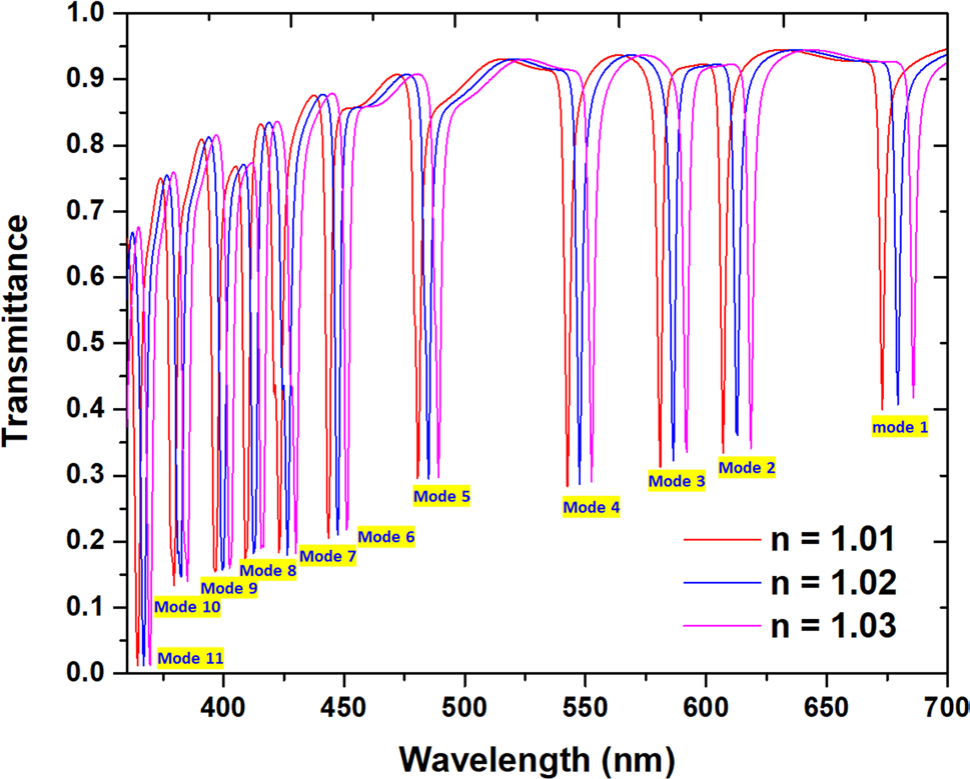
Transmittance spectra of eleven channels (i.e., modes 1–11) in the proposed color filter with the ambient media, *n*, are 1.01, 1.02, and 1.03, respectively.

The proposed color filter can function as a RI sensor when the structure involves the detecting medium, i.e., the *n*_eff_ in the bus waveguide and six circular cavities is changed and is exceptionally responsive to the surrounding material. Figure 6 shows the transmittance spectra of the proposed color filter with the ambient media, *n*, are 1.01, 1.02, and 1.03, respectively. As observed, the transmission dips show a redshift as the increasing RI and a linear relationship between the *n*_eff_ and the λ_res_, which well agrees with Eq. (2). The sensitivity's increment is closely related to the SPR and CPR effects in the color filter, leading to an interaction with the variation of the surrounding medium^67^.

**Figure 7:**
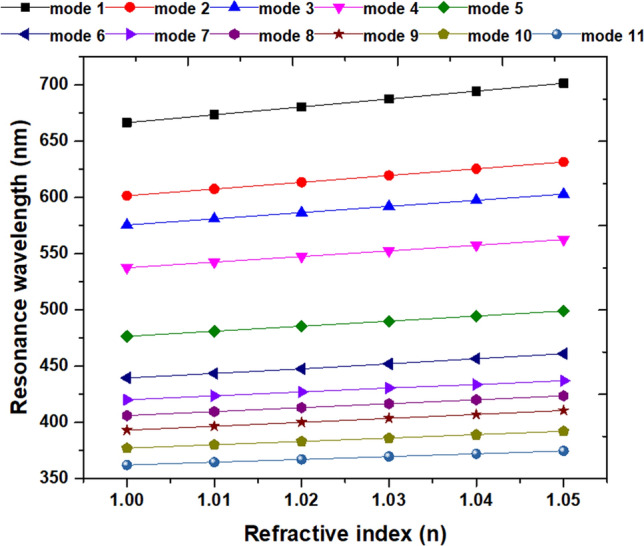
Calculated resonance wavelength (λ_res_) from mode 1 to mode 11 for the proposed color filter versus the RI with the filling media, *n*, in the range of 1.01–1.05 in the step of 0.01.

An excellent RI sensor should have the features of the high value of sensitivity (S), figure of merit (FOM), Q-factor, and dipping strength (∆D). Figure 7 depicts the calculated λ_res_ from mode 1 to mode 11 for the proposed color filter versus the RI with the surrounding media, *n*, in the range of 1.01–1.05 with an increment of 0.01. We outline the S, FOM, Q factor, and ∆D of the proposed color filter from mode 1 to mode 11 in Table 2. Note that the maximum values of S, FOM, Q factor, and ∆D obtained from mode 1 to mode 11 can achieve 700 nm/RIU, 350 1/RIU, 349.0, and 65.04%, respectively, reveal distinguished sensor properties of the proposed color filter. These values are more remarkable than the previous articles (e.g.,^37,42^) and show multiple channels of the color filter and RI sensor functions that can match the requisite wavelength of ultraviolet and visible. We compare the designed structure with the published similar systems in Table 3. Besides, we can vary the desired working wavelengths by optimization of structural parameters. The other RI values has the same trend as the obtained results from Fig. 7.

**Table 2:** The S, FOM, Q factor, and ∆D of the proposed color filter from modes 1 to 11.

Mode	1	2	3	4	5	6	7	8	9	10	11
S (nm/RIU)	700	600	550	500	450	400	350	350	350	300	250
FOM (/RIU)	350.0	300.0	275.0	250.0	225.0	200.0	175.0	175.0	175.0	150.0	125.0
Q factor	349.0	315.0	301.2	281.3	248.6	229.5	218.5	211.0	204.6	195.3	187.3
∆D (%)	53.28	57.66	63.00	65.01	62.54	66.71	64.85	58.70	65.89	60.39	65.04

**Table 3:** Comparison of the proposed optical filter with the previous similar color filter structures.

References	Number of channels	Max. S (nm/RIU)	FOM (1/RIU)	∆D	FWHM (nm)
^37^	6	608	57.53	85.00	10.56
^68^	1	–	16.70	70.00	76.00
^69^	9	–	156.94	30.00	150.00
^38^	3	–	–	40.00	150.00
^42^	6	700	191.6	61.00	40.00
^29^	1	–	159.6	65.00	15.00
^70^	4	–	–	40.00	60.00
This work	11	700	255.56	81.89	2.00

## Application as an optical switch

Optical switching is a vital device for applying in IOCs^71,72^. Recently, some all-optical switches based on MIM waveguides have been developed and investigated since the features of strong light confinement and electromagnetically induced transparency (EIT) spectra^73–75^. This kind of all-optical switch could overcome diffraction limits in nano-scale dimensions. Liu et al. proposed an ultrafast and low-power all-optical switch with femtosecond-scale feedback time using a MIM waveguide with a slot^73^. Negahdari et al. designed an all-optical plasmonic switch based on a MIM split square ring resonator with the reverse and direct switching operations^74^. Bazgir et al. used MIM-based dumbbell-shaped cavity slots by exploiting a DNA composite and claimed the switchable DNA element can manipulate the electric field and transmission line shape^75^. The sharp transmittance line shapes with high FOM, Q-factor, and dipping strength obtained in the proposed color filter are workable for constructing optical switches. In Fig. 2, the transmittance curve displays a steep decline from transmittance peak (“on”) to transmittance dip (“off”) with a small FWHM, and thus can utilize this property to establish an optical switch device. The transmission contrast ratio can express as^76^

4 $$ {\text{M}}_{{{\text{ext}}}} = { 1}0{\text{ log }}\left( {{\text{T}}_{{{\text{max}}}} /{\text{T}}_{{{\text{min}}}} } \right) \, \left( {{\text{dB}}} \right) $$

where T_max_ and T_min_ represent “on” (peak) and “off” (dip), respectively. Based on Eq. (4), we have a maximum extinction ratio of 174.50 dB and summarize the extinction ratio values of the proposed color filter in Table 4. These values are great higher than the previous literature in the wavelength range of ultraviolet and visible (e.g.,^71,76–78^).

**Table 4:** The extinction ratio values of the proposed color filter from mode 1 to 11.

Mode	1	2	3	4	5	6	7	8	9	10	11
M_ext_ (dB)	37.09	42.53	48.46	50.82	50.79	62.48	65.17	63.25	73.97	72.05	174.50

## Application for inspecting hemoglobin concentration in human blood samples

The proposed color filter can also detect biological fluids, e.g., hemoglobin concentration (HC), since its suitable wavelength range with multiple resonance modes and excellent sensing performance^79^. For application, six cavities and the bus waveguide can be used as the resonator and then the blood specimen placed inside them. In a practical situation, the blood sample can drop into the air regions of six circular-shape cavities by capillary attraction^80–82^, and the bus waveguide with a small amount due to the SPPs are very sensitive to the variation of the ambient material^83,84^. Several research groups experimentally and theoretically investigated the RI of blood groups (O, A, and B) at visible and near-infrared wavelengths (380–1100 nm) for diverse blood specimens^85–87^, in which the operation wavelengths quite fit our proposed color filter.

The relationship between the blood samples’ RI and HC can signify by^88^

5 $$ n_{b} = n_{0} + \alpha C, $$

where *n*_0_ is the blood sample’s effective RI when C = 0. *C* is the blood sample’s HC (in g/L), and *α* is the specific refraction increment fixes a particular blood group. Besides, the temperature T (in the Kelvin unit) and the operating wavelength can also influence the value of *n*_*b*_. As a result, Eq. (5) can express by^81,84^:

6 $$ n_{b} = n_{0} + \alpha C + \beta T + \delta \lambda + \sigma \lambda^{{2}} + \gamma \lambda^{{3}} , $$

where *α*, *β*, *δ*, *σ*, *γ* are Cauchy coefficients that vary with blood group, and these coefficients can obtain from Ref.^80^.

**Figure 8:**
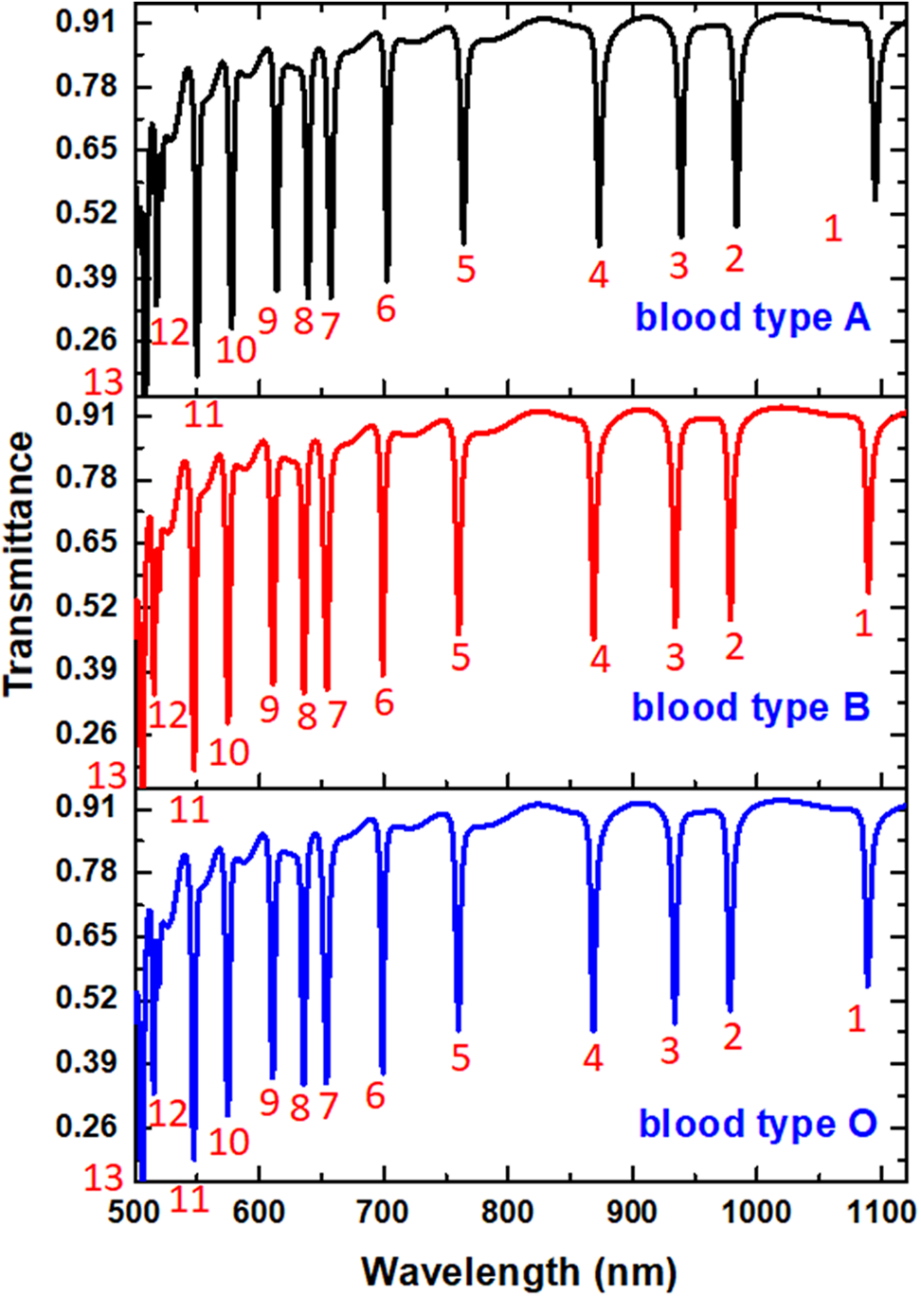
Transmittance spectrum of three different blood groups (O, A, and B) for CA = 146.2 g/L, CB = 111.1 g/L, and CO = 108.9 g/L at 300 °K put in the proposed color filter.

**Figure 9:**
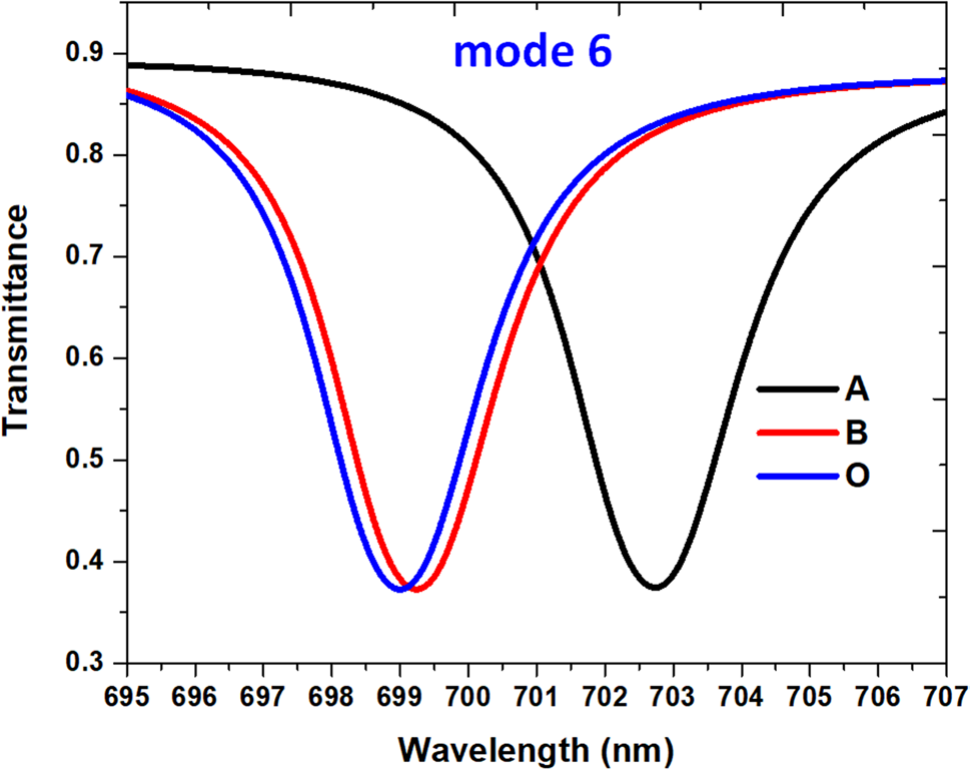
Illustration of the transmittance spectrum of mode 6 in the wavelength of 695–707 nm.

Based on the calculation results from Eq. (6), the HC of different blood group specimens in Ref.^89^ for temperature at T = 300 °K is found as C_A_ = 146.2 g/L, C_B_ = 111.1 g/L and C_O_ = 108.9 g/L. Figure 8 depicts the transmittance spectrum of three different blood groups (O, A, and B) for C_A_ = 146.2 g/L, C_B_ = 111.1 g/L, and C_O_ = 108.9 g/L in room temperature (300 ^°^K) put in the proposed color filter. The geometrical parameters are the same as used in Table 1. As shown in Fig. 8, we have thirteen channels (labeled by number) in the 500–1120 nm wavelength. These ranges can shift from the ultraviolet and visible light spectra blend into the near-infrared range. Compared to its counterpart without solution (see Fig. 2), the two more working channels redshift from the shorter wavelengths because of the increase of the *n*_eff_ of HC specimens (see Eq. (2)). Using the recently introduced detectors can achieve a λ_res_ shift of as small as 0.1 nm. We illustrate the transmittance spectrum of mode 6 in the wavelength of 695–707 nm as an example (see Fig. 9). The λ_res_ for A, B, and O-groups are 702.74 nm, 699.24 nm, and 699.00 nm, respectively. Note that a very narrow FWHM (~ 2 nm) is attainable in each curve.

**Figure 10:**
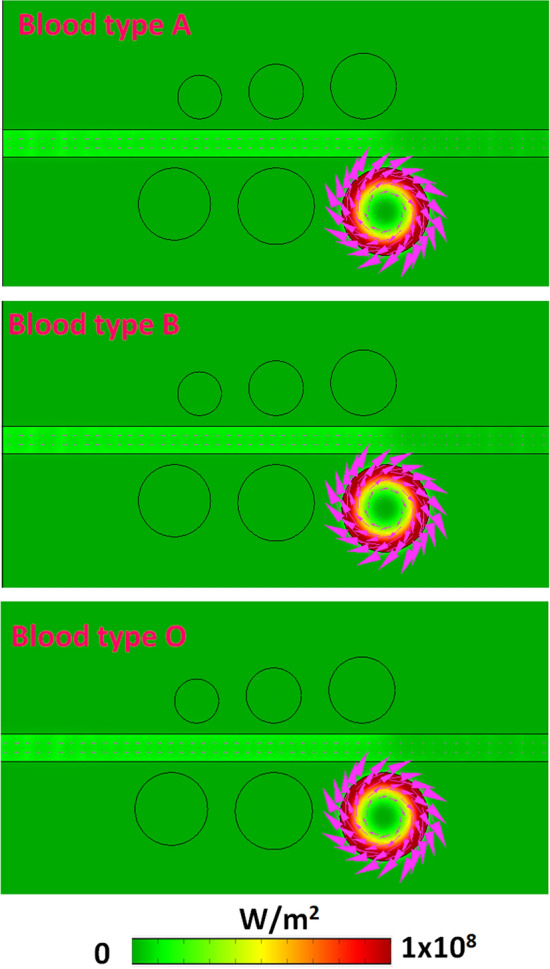
Time average power flow (W/m^2^) with arrows of three blood types of hemoglobin concentrations, i.e., 146.2 g/L, 111.1 g/L, and 108.9 g/L, for mode 6.

Going further into their mechanism, Fig. 10 shows the time average power flow (W/m^2^) with arrows of three blood types of HCs, i.e., 146.2 g/L, 111.1 g/L, and 108.9 g/L, for mode 6. As observed, the power flows well tripped in the right side of the bottom cavities. Significantly, the high-density energy flows appear in the interface between the bus waveguide and resonators. Besides, the dense energy flows propagate on the cavity's circumference surface and efficiently connect to the bus waveguide, showing a better coupling effect between the bus waveguide and cavities. As the three blood types of HCs are involved in the plasmonic filter system, leading to increased local environmental RI, the λ_res_ will redshift since the reduced repulsion between dipoles (i.e., positive–negative pairs) with the same orientation, thus reducing the energy of the plasmon oscillations. It is important to note that most of the power flows in each case formed a ring-like spot on the cavity's periphery surface and propagated in a clockwise direction. These results show that the measurement of HCs with the proposed color filter can be conducted conveniently with high accuracy at the desired wavelength, and the proposed design can apply in diverse biomedical and RI sensing applications.

**Figure 11:**
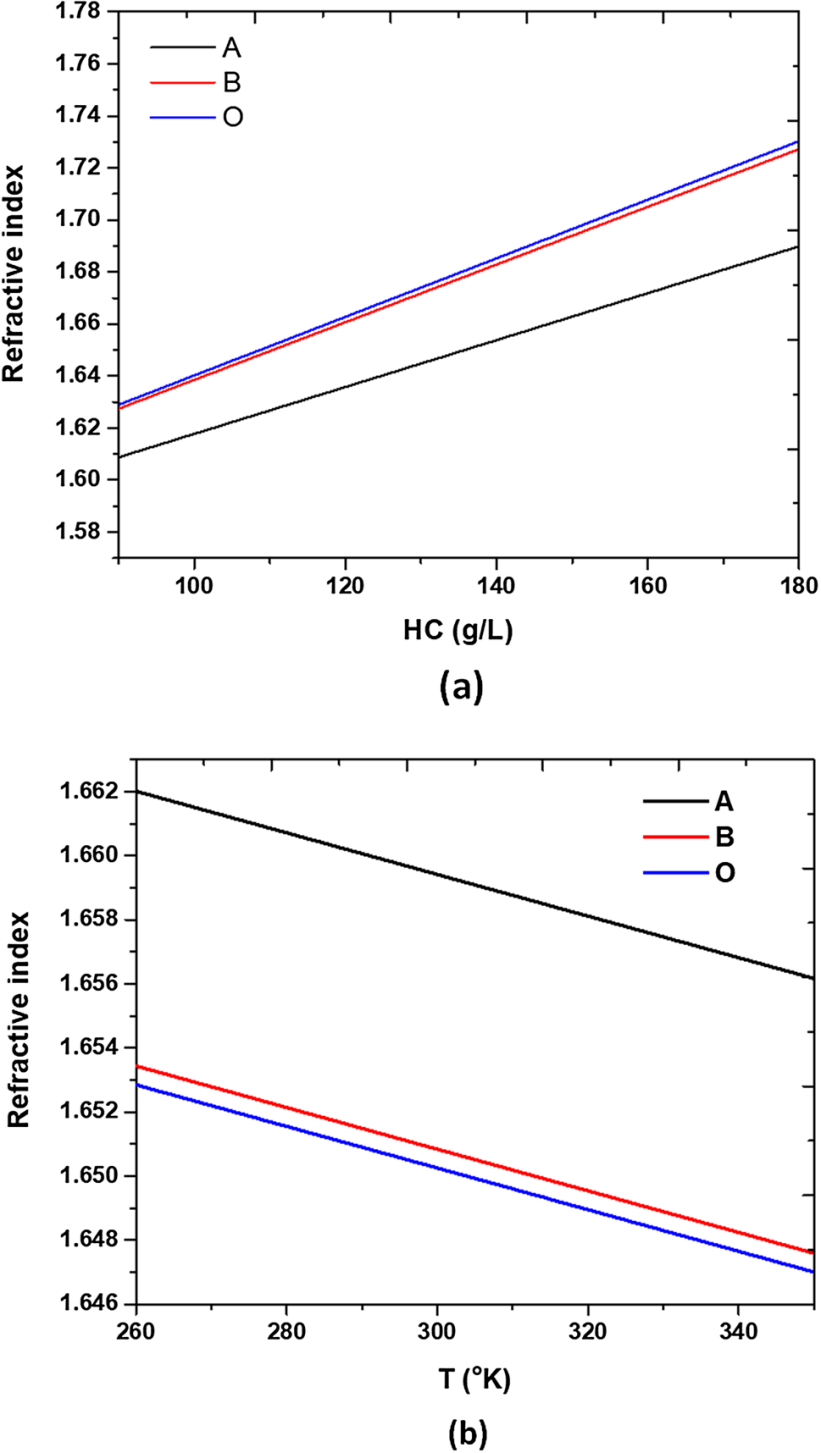
(**a**) Three blood types of refractive indices spectra versus hemoglobin concentration ranging in 90–180 g/L for a selected working wavelength, λ = 700 nm, at T = 300 ^°^K. (**b**) Three blood types of refractive indices spectra versus the temperature in the range of 270–330 °K with C_A_ = 146.2 g/L, C_B_ = 111.1 g/L, and C_O_ = 108.9 g/L for λ = 700 nm.

According to Eq. (6), parameters such as HC and temperature closely relate to the RI variation of different blood groups. In addition, oxygenated hemoglobin has a more negligible light absorbance at λ = 700 nm than deoxygenated hemoglobin; thus, we examined the incident wavelength of λ = 700 nm. Figure 11a plots the RI variations corresponding to the three blood types within the HC ranging in 90–180 g/L for a selected working wavelength (λ = 700 nm) at the temperature of T = 300 K. As seen, the RI of the three blood types increases linearly with the increase of HC. As a result, one can get the related HC if the RI can attain through the SPPs modes inspection. Figure 11b also reveals the varying RI with temperature T in the range of 260–350 ^°^K at λ = 700 nm when the HCs chose C_A_ = 146.2 g/L, C_B_ = 111.1 g/L and C_O_ = 108.9 g/L, respectively. In Eq. (6), the parameter T (temperature) closely relates to the RI variation of different blood groups, and T has less influence on the dimensions of the circular cavities and the air gap in the proposed MIM waveguide system^80–82^. As seen in Fig. 11b, the growth trend of the line profiles of RI versus T also displays a linear relationship between RI and T, and as the T increases, the RI decreases. Therefore, the transmittance dip (i.e., λ_res_) will blueshift as T increases because of the decrease of effective refractive index (RI) (see Eq. (2)). Compared with other reported devices for HC detection, the performance of our proposed color filter possesses novel features and flexible applications in the wavelength range of 350–1100 nm and has the advantage of multiple channels and the functions of RI sensors and optical switches.

## Conclusion

In summary, we propose a multichannel color filter capable of filtering out eleven modes, including individual colors in red, orange, yellow, green, blue, four violets, and two ultraviolets in the wavelength range of 350–700 nm. The designed structure consists of one MIM bus waveguide side-coupled to six circular cavities. We performed the simulations by using FEM to investigate the transmittance spectrum and EM wave distributions in detail. Such filter possesses the other two functions, including RI sensor and optical switch, meantime has an ultracompact scheme, less structural parameters, and small footprint, benefiting from the independent tunability of different colors. The maximum values of sensitivity, figure of merit, quality factor, dipping strength, and extinction ratio can reach 700 nm/RIU, 350 1/RIU, 349.0, 65.04%, and 174.50 dB, respectively, which reveal the excellent functions of sensor performance and optical switch. This sensor can widely be used in gas and biochemistry since its ease of preparation, excellent sensing performance, and broad working wavelengths with multiple modes, suitable for detecting gases and fluids (e.g., hemoglobin concentration). This research can receive pivotal applications on highly IOCs.

## Data Availability

The authors declare that all data supporting the findings of this study are available from the corresponding author upon reasonable request.
